# Experimental Investigations of Reinforced Concrete Beams with Innovative Truss-Shaped Reinforcement System

**DOI:** 10.3390/ma14071652

**Published:** 2021-03-27

**Authors:** Adam Stolarski, Jacek Zychowicz

**Affiliations:** Faculty of Civil Engineering and Geodesy, Military University of Technology, 2 gen. Sylwestra Kaliskiego Street, 00-908 Warsaw, Poland; jacekzychowicz@gmail.com

**Keywords:** reinforced concrete beams, truss-shaped reinforcement system, experimental investigations

## Abstract

The purpose of the work is an experimental analysis of the behavior of reinforced concrete beams with a new, patented system of truss-shaped reinforcement. Experimental tests of reinforced concrete beams with conventional reinforcement and with truss-shaped, mass equivalent reinforcement, with two different values of longitudinal reinforcement ratio, were carried out. The testing results of the load-carrying capacity and displacements of beams are presented. The cracking and failure mechanism of beams with a new truss-shaped reinforcement system was also analyzed. The test results for conventionally reinforced beams and with truss-shaped reinforcement were compared. The test results show that the use of the truss reinforcement has an influence on increasing the load-carrying capacity of beams. The amount of this increase depends on the total longitudinal reinforcement ratio and reaches as much as 95% for beams with a low reinforcement ratio and 12% for beams with a higher reinforcement ratio. Based on the investigation of the cracking mechanism, it can be concluded that the failure of the beams with transverse truss-shaped reinforcement occurs with a greater number of smaller cracks, which are more evenly distributed along the length of the cracking zone, and have a shorter range over the cross-section depth, which results in their smaller opening widths. The comparative analysis shows the effectiveness of the proposed reinforcement system, justifying the high potential possibilities of its use for the reinforcement of concrete structural elements.

## 1. Introduction

### 1.1. General Characterization of the Reinforcement of Reinforced Concrete Elements

Concrete structures reinforced with inserts made of high-strength materials are the most commonly used in the building structures of the world.

The development of reinforced concrete began with the commonly known inventions of Joseph-Louis Lambot, Joseph Monier, William Boutland Wilkinson, and Francois Hennebique in the nineteenth century. In turn, the development of concrete prestressing technology in the early twentieth century was initiated by Eugène Freyssinet.

In the pioneering invention of Khan [1], a reinforcement system called “Khan Trussed Bar System” was presented. Khan, noticing that concrete has a high compressive strength and a low tensile strength, conducted scientific engineering experiments and created the concept of reinforcement for reinforced concrete elements, with bent bars attached to the longitudinal reinforcement at an angle of 45 degrees. The features and application of this reinforcement system have been described by the Trussed Concrete Steel Company [2] and e.g., in the review paper by Salmon and Elliott [3].

The current rules for the use of classic reinforced concrete elements in the form of main bars parallel to the middle plane and auxiliary bars essentially located perpendicular to the main reinforcement are well-established and known thanks to the publications of The International Federation for Structural Concrete (fib), e.g., Walraven et al. [4] or American Concrete Institute, e.g., Taylor et al. [5,6]. These principles have also been described in detail, among others, in the works of Polish authors Kobiak and Stachurski [7,8,9,10], Knauff [11], and Starosolski [12].

These principles are used and developed in many studies of reinforced concrete elements. These studies concern both different types of reinforced concrete elements as well as experimental and computational research methods.

Tests of traditional structural elements reinforced with steel bars of the main reinforcement in tension and with usual stirrups in shear are the subject of many works. However, modern research on reinforced concrete beams focuses on the material modification of concrete and the material diversity of reinforcing bars.

An experimental and a numerical study were carried out on the flexural crack behavior of reinforced concrete beam members by Sandeep Das et al. [13]. The experimental investigation was focused on the effect of flexural crack by varying the percentage of tensile steel on beam sections. A computer vision-based data-driven numerical tool for crack representation and quantification was developed based on the real-time video surveillance data of the flexural testing on beams.

A study on the influence of shape memory alloys (SMA), glass fiber-reinforced polymer (GFRP), and steel longitudinal rebars on flexural and shear behavior of reinforced concrete beams was presented by Karimipour and Edalati [14]. The results indicated that using GFRP and SMA rebars improves maximum bending capacity and deformation, and prevents the rapid reduction in the bearing capacity after the maximum load point of beams.

An experimental investigation into the effect of GFRP needles as coarse aggregate partial replacement in concrete on the shear behavior of large-scale reinforced concrete beams was performed by Nie et al. [15]. The GFRP needles were obtained by cutting FRP waste into short-length randomly distributed reinforcing bars. An enhancement in the load-carrying capacity was observed in beams with helically wrapped needles, while beams with smooth needles showed a slight reduction in the load-carrying capacity. The presence of GFRP needles significantly increased the amount of total energy absorbed by the beams.

The concrete beams were tested to investigate the flexural performance of concrete beams reinforced with three different reinforcement bar type (hybrid, GFRP, and steel) and the five different reinforcement ratios by Said et al. [16]. The test results showed a significant enhancement in the maximum load-carrying capacity due to increasing the hybrid reinforcement ratio.

Research to evaluate the structural performance of two-layer fiber-reinforced concrete beams with glass fiber-reinforced polymer (GFRP) and steel rebars under quasi-static loads was carried out by Nematzadeh and Fallah-Valukolaee [17]. The results showed that adding fibers to the compression zone of the section led to a higher ductility in both GFRP rebar and steel rebar reinforced beams, while adding fibers to the tensile zone led to a higher ultimate flexural strength. An increase in the ratio of GFRP and steel reinforcement together with a greater concrete compressive strength in the layered beams enhanced their flexural performance in terms of load-carrying capacity, flexural stiffness, and ductility. Replacing steel rebars with GFRP ones led to a decrease in these parameters.

Almost all above mentioned experimental tests were carried out using point-contact measurement devices, i.e., clip strain gauges and linear variable differential transducers.

Research is being carried out on elements reinforced with traditional steel reinforcement surrounded by a concrete matrix additionally reinforced with a system of steel fibers or a mixed system of steel, polypropylene, or glass fibers. Smarzewski presented research on slabs, beams, and deep beams with/and without openings made of high-performance concrete and hybrid (steel and polypropylene) fiber reinforced high-performance concrete [18,19,20]. In these studies, the non-contact, three-dimensional, deformation measuring system ARAMIS [21] was effectively used.

In terms of research methods, the non-contact methods of recording the results and measurements of displacements and deformations are increasingly used. Liu et al. proposed a framework to optimize two-dimensional measurements in concrete structure models with a digital image correlation (DIC) system at different orders of accuracy [22]. A good example of using the DIC method is the work of Funari et al. [23]. With regard to reinforced concrete elements, the description and application of the DIC method has been systemically presented by Skarżyński et al. [24]. The application of DIC has been practically used in investigations of size effect in reinforced concrete beams with longitudinal steel and basalt bars but without shear reinforcement by Syroka-Korol and Tejchman [25]. The DIC technique was also applied by Suchorzewski et al. to visualize strain localization on the concrete surface of the longitudinally reinforced concrete beams with separately varying height and length, in order to investigate the size effect on nominal strength and post-critical brittleness [26].

In the literature, modifications of the transverse shear reinforcement replacing usual stirrups in reinforced concrete beams are not often found.

However, there are examples of entwined reinforcement, also known as laced reinforcement. This reinforcement is used in reinforced concrete elements and is specially called laced reinforced concrete (LRC), Anandavalli et al. [27]. In this paper, an approach for finite element modeling of RC/LRC structural elements that are primarily under flexure was proposed. The approach considered RC/LRC as a homogenous material whose stress-strain characteristics were derived based on the moment-curvature relationship. Numerical studies on LRC beams were carried out and the results was compared with those of the experimental values.

Entwined reinforcement is also used in the laced steel–concrete composite (LSCC) system, wherein two outer steel overlays on concrete are joined by steel laced bars and horizontal cross bars without welding, Anandavalli et al. [28]. This paper presents the experimental investigations carried out on two beam specimens: one with 45° lacing and another with 60° lacing. The loading was conducted under displacement control mode. Experimental results indicate that both types of the beams exhibit almost similar strength performance, while the one with 60° lacing performed better in terms of deformation.

The use of continuous spiral reinforcement has been examined in RC elements with rectangular cross-sections by Karayannis and Chalioris [29]. Test results indicated that the use of continuous rectangular spiral reinforcement (known as normal formation of spirals) and rectangular spiral reinforcement with shear-favorably inclined links (known as advanced formation of spirals) caused enhanced bearing capacity and shear performance in the beams in comparison with results for beams with usual stirrups.

An experimental study on assessing the possibility of obtaining more ductile shear failures using a Ni-Ti alloy spiral reinforcement was presented by Mas et al. [30]. It was shown that the Ni-Ti spiral reinforcement makes it possible to obtain highly deformable concrete elements even for beams failing in shear.

The presented literature review shows that (to the best of the authors’ knowledge) geometrical shear reinforcement arrangements based on the permanent connection of crossed transverse bars, which can be an alternative to the conventional reinforcement arrangement, have not been presented in the literature so far.

This fact derived the authors’ motivation and strive to design the more homogeneous structural elements as concrete material compositions reinforced with special arrangements of reinforcing steel.

Such a goal is met by the innovative truss-shaped reinforcement arrangement of reinforced concrete elements proposed in this paper.

### 1.2. The Proposed Truss-Shaped Reinforcement System

**Figure 1:**
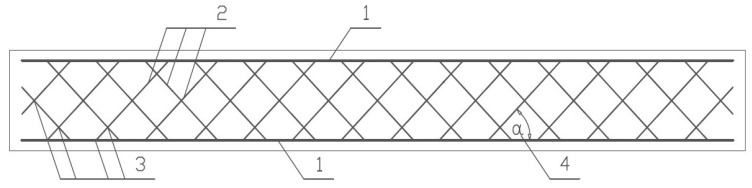
Schema of the truss-shaped reinforcement system of a beam: 1—longitudinal bars (chords), 2—diagonal transverse bars (cross braces), 3—joints of bonding (welding or resistance welding), 4—cross braces inclination angle 30°–60°.

The subject of this paper is a new type of concrete reinforcement in the form of plane meshes with a truss arrangement of bars (Figure 1), the solution of which was patented by Stolarski and Zychowicz [P1].

The innovation of the proposed system of plane meshes for concrete reinforcement with a truss bar arrangement is specific in the following features:

1. Mesh arrangement of steel bars consists of longitudinal bars—chords (1) and repeatedly crossed diagonal transverse bars—cross braces (2).
2. All the truss bars are durably connected at the joints (3) by bonding (welding or resistance welding) in a hyperstatic (over-stiffened) truss system.
3. Transverse bars—cross braces—are arranged in two directions at an angle α from 30° to 60° (4) in relation to the longitudinal bars.

The truss-shaped reinforcement is self-supporting even before concreting. Using the self-supporting truss reinforcement, much higher load-carrying capacities are obtained than using the conventional type of reinforcement with the use of the same amount of steel.

The truss arrangement of bars increases the homogeneity ratio of the concrete-steel composition and thus strengthens the reinforced concrete elements not only in the places where diagonal cracks occur, but also in the entire element.

Due to the homogenization of the concrete-steel composition by the use of a system of flat meshes with the truss arrangement of reinforcing bars, the stresses are more evenly distributed in the element, and the failure mode occurs as a result of the appearance of many cracks with relatively small widths, dispersed at greater distances from the points of load application.

The anchoring conditions of the crossed truss bars in concrete improved in the places of the welded joints. However, the required anchorage lengths in accordance with the standard requirements should be used. The spacing of the diagonal transverse bars should be at least 3 times the maximum size of the aggregate used for the concrete.

Connections between structural elements should be designed using the principle of reinforcement continuity of each element and taking into account the principle of mutual interpenetration of meshes. In critical joints places, the welded joints of reinforcing bars can also be used in accordance with the standard requirements.

As described in Figure 1, the angles between the transverse bars of 30° to 60° should be applied depending on the cross-sectional height.

The arrangement of the transverse bars (cross braces) of the reinforcement mesh at a different angle also allows for the optimization of the strengthening of the structure in the most stressed places. The application of the same configuration of the slope angle of the transverse bars throughout the structural element enables this element to be adapted to the variable system of external loads. In contrast, it is possible to differ the configuration of the slope angle of the transverse bars along the length of the element in a situation of ensuring a fixed and time-invariant system of external loads.

The application of the proposed reinforcement system may be limited by:

- the need to prefabricate the reinforcement meshes,
- the need for multi-spot welding that is effective only in industrial conditions, and
- the need for precise design and realization of connection of various structural elements from prefabricated reinforcing meshes.

These limitations can be significantly minimized in the perspective of the development of automation of the reinforcement mesh arrangement fabrication, even to the level of the “printing” technology of flat and spatial reinforcement. Thus, it would be possible to take advantage of the qualitative advantages of the proposed reinforcement arrangement leading to a reduction in the cost of manufacturing and transportation.

The work of Stolarski and Zychowicz [31] presents the results of preliminary tests of beam models with a span of 1.0 m and a relatively small cross-section of 0.1 m × 0.15 m. This article presents the results of experimental tests and calculation analyses of concrete beams reinforced with truss mesh in accordance with Figure 1 and comparison with the test results of conventionally reinforced beams. Preliminary tests of beams with this reinforcement showed lower deflections, higher load-carrying capacities, and more uniform and dispersed cracking compared to conventionally reinforced elements, with the equivalent reinforcement in terms of the weight of the reinforcement used. These facts indicated an increase in the degree of homogeneity of reinforced concrete beams with the new reinforcement arrangement.

### 1.3. The Aim and the Scope of the Paper

As the properties of the elements observed on short beams may not fully reflect the actual work of the elements, the aim of this paper is an experimental comparative analysis of the behavior of reinforced concrete beams with a truss-shaped system of transverse reinforcement bars, with a span of 3 m.

Experimental tests of reinforced concrete beams with conventional reinforcement and with appropriately mass equivalent truss-shaped reinforcement, each with a different reinforcement ratio of longitudinal reinforcement, were performed. It was assumed that the reinforcement mass balance determines the equivalence of conventional and truss reinforcement.

The beams were investigated until failure in a four-point flexural test. The results of testing the load-carrying capacity and displacement of beams are presented. The non-contact digital image correlation method was used to measure the displacements.

The cracking and failure mechanism of beams with a new truss-shaped reinforcement system was also analyzed. The images of the crack pattern are presented without measuring the crack width, but with visualization of the cracks’ range at the height of the cross-section.

The test results for conventionally reinforced beams and with truss-shaped reinforcement were compared. The effectiveness of the truss-shaped reinforcement system was indicated.

## 2. Subject of Research—Reinforcement Systems in Concrete Beams

Reinforced concrete beams with a support span of 3.0 m were tested. The beams were loaded in a four-point pattern.

The beams with double, symmetrically reinforced, rectangular cross-sections were tested. Two types of beams were compared: beams reinforced with conventional reinforcement (***C***) and with truss reinforcement (***T***). For each type of beam, two series of beams were tested with the use of reinforcement systems with different rates of longitudinal reinforcement.

**Figure 2:**
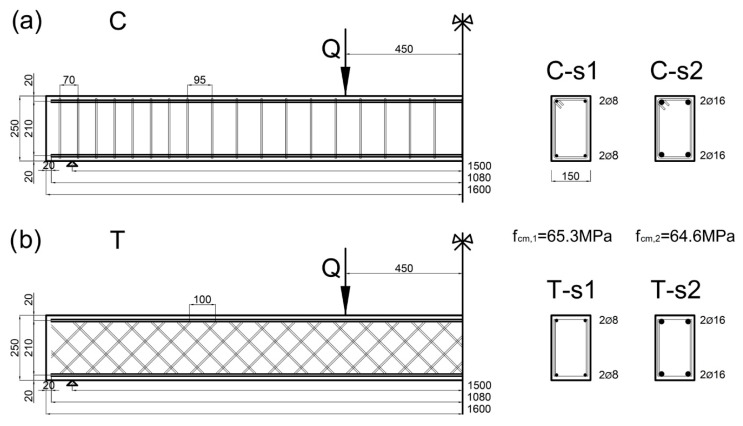
Schemes of beam reinforcement: (**a**) conventional layout of Series 1 ***C-s1*** and Series 2 ***C-s2***, (**b**) truss-shaped layout of Series 1 ***T-s1*** and Series 2 ***T-s2***.

The descriptions of geometry and the reinforcement layouts of the beams are shown in Figure 2.

In the Series 1 (***s1***) beams, the longitudinal ribbed bars with a diameter $\emptyset_{l1}=8\;\mathrm{mm}$ were used and in the Series 2 (***s2***) beams—with diameter of $\emptyset_{l2}=16\;\mathrm{mm}$. The total ratio of longitudinal reinforcement in the beams ***s1*** is $\rho_{l1}=2\;\times \;0.291=0.582\%$, and in the beams ***s2***—is $\rho_{l2}=2\;\times \;1.166=2.332\%$. Transverse reinforcement in the form of double-arm stirrups in ***C***-type beams and in the form of two flat trusses in ***T***-type beams were made of ribbed bars with a diameter of $\emptyset_{t}=6\;\mathrm{mm}$.

**Figure 3:**
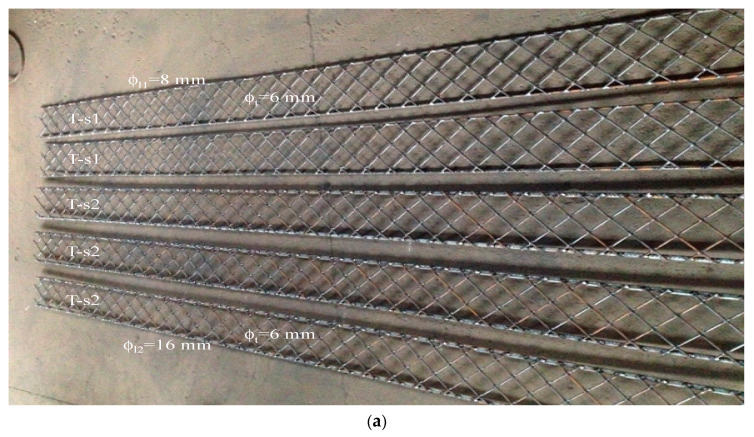

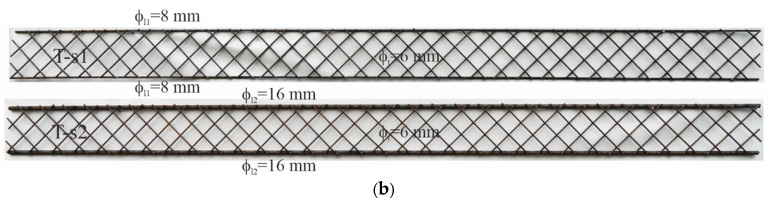
Plane meshes for the truss reinforcement of beams Series 1 ***T-s1*** and Series 2 ***T-s2***: (**a**) general view, (**b**) transverse view.

The actual layout of the truss-shaped reinforcement of the beams Series 1 ***T-s1*** and Series 2 ***T-s2*** is shown in Figure 3.

## 3. Test Methods

**Figure 4:**
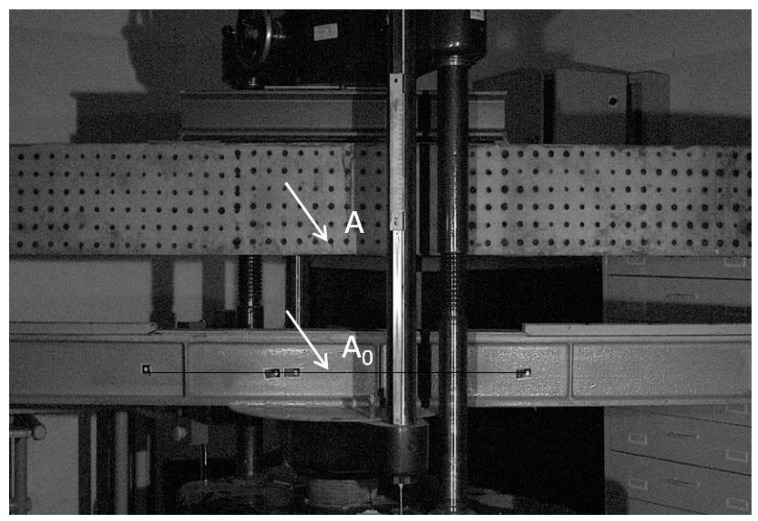
Measurement points for displacements *A* in relation to the reference point *A*_0_.

Measurement points for observation and recording of displacements were marked on the front surface of the beams. In order to determine the deflection, the displacement of the point at the place of the expected maximum beam displacement (at the lowermost measurement point in the symmetry axis) was analyzed in relation to the reference point on the steel base of the beam supports, Figure 4.

The load was applied with a constant increase in force over time of 1 kN/s. The tests were performed up to the value of the failure load of the beams.

The course of the study was recorded using the Phantom MIRO M310 camera with a recording speed of 3260 frames per second (fps) at a maximum resolution of 1280 × 800 pixels and a maximum speed of 650,000 fps, with a minimum resolution of 64 × 8 pixels.

The registration of the results at particular time instants made it possible to determine the displacements of the measurement points in relation to the reference point in the {*X*, *Y*} coordinate axes of the image. The displacement values read in pixels were then converted to displacement measurement units in millimeters. The pixel size has been converted to displacement measurement units using the standard pixel dimension.

The range of cracks along the height of the cross-section was estimated based on optical analysis of the recorded images at selected time instants. The accuracy of the estimation results from the resolution of the recorded image. This fact affects the possibility of determining the time of the appearance of the first crack defining the cracking force and, to a lesser degree, of determining the range of the cracks, and practically makes it impossible the determination of crack occurrences with a width smaller than the pixel size.

Based on the recording of the beam testing process, the following parameters and phenomena were recorded:

1. Load and deflection in the time function, allowing to determine the dependence of the load as a displacement function.
2. The order of appearance, number, and location of cracks in the load function, allowing to determine cracking and the failure mechanism of the beam.

As part of the experiment, strength tests of the construction materials were also carried out: compressive strength of concrete and tensile strength of reinforcing steel bars.

The beams of Series 1 and Series 2 were made of two lots of concrete mixes of the designed class C50/60. The beams and the material testing were made in accordance with the requirements of standard EN 206:2016 [32] and related standards [33,34].

Before concreting the beams, the consistency of the concrete mixture was tested using the falling cone method. Concrete samples with the declared dimensions of 150 × 150 × 150 mm were made. The samples were stored after demolding in a chamber with humidity above 95% and temperature 20 ± 2 °C.

The compressive strength test of the concrete was performed on the CONTROLS I class testing machine.

Samples of ribbed steel bars used for reinforcement of the beams were tested. Stress-strain diagrams with a clear yield limit were obtained for all bars. As a yield limit $R_{e}$, the upper yield limit was adopted $R_{eH}$. The tensile strength of the bars was also tested $R_{m}$.

## 4. The Test Results of Structural Materials

### 4.1. Concrete

The results of compressive strength tests of concrete samples $f_{ci}$, mean compressive strength $f_{cm}$, with an indication of the minimum compressive strength $f_{c,min}$, are shown in the Table 1.

Based on the compliance criteria for initial production and the test results given in Table 1, the compressive strength of the concrete for both series of beams was found to be in accordance with the strength class C50/60.

### 4.2. Reinforcement Steel Bars

Reinforcing steel of grade B500SP with the increased ductility of class C was used (see: www.epstal.pl accessed on 18 March 2021). Table 2 shows the results of the reinforcement steel bars tensile strength tests.

## 5. The Test Results of Load-Carrying Capacity and Displacements of Beams

**Figure 5:**
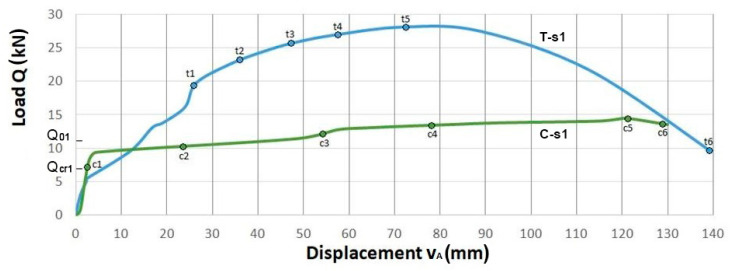
Displacement of the measuring point A in the midspan of beams Series 1 with the proposed truss reinforcement ***T-s1*** and with conventional reinforcement ***C-s1***.

**Figure 6:**
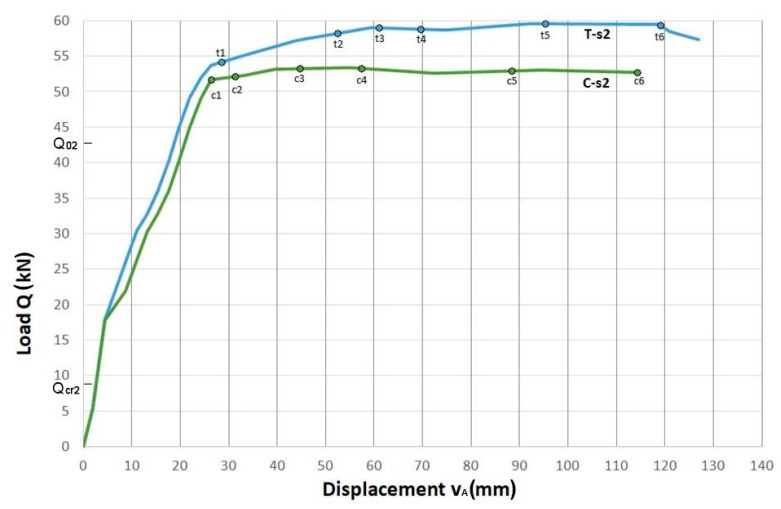
Displacement of the measuring point A in the midspan of beams Series 2 with the proposed truss reinforcement ***T-s2*** and with conventional reinforcement ***C-s2***.

Diagrams of beam displacements in the load function for two series of beams with conventional reinforcement and with truss reinforcement are presented in Figure 5 for beams of Series 1 and in Figure 6 for beams of Series 2.

The diagrams show the analytical values of the cracking force $Q_{cr}$ and the failure force $Q_{0}$. These forces were determined for the bending moments determined for the double, symmetrically reinforced cross-section, respectively for the elastic phase *Ia* and for the limit phase *III*, see Appendix A. The values of these forces are as follows:

(1) $$\mathrm{for}\;\mathrm{beam}\;\mathrm{of}\;\mathrm{Series}\;1:\;Q_{cr1}=6.87\;\mathrm{kN}\;\mathrm{and}\;Q_{01}=10.88\;\mathrm{kN},$$

(2) $$\mathrm{for}\;\mathrm{beam}\;\mathrm{of}\;\mathrm{Series}\;2:\;Q_{cr2}=8.00\;\mathrm{kN}\;\mathrm{and}\;Q_{02}=42.63\;\mathrm{kN}.$$

The selected points of ${\left(Q,v_{A}\right)}_{i}$ coordinates are indicated on each graph, for which the images of cracking state were presented in a further part of the paper.

In beams of Series 1 with a low total longitudinal reinforcement ratio of $\rho_{l1}=0.582\%$, the beam with truss reinforcement ***T-s1*** showed an increase in the load-carrying capacity by 95% compared to that for the beam with conventional reinforcement ***C-s1***.

In turn, in beams of Series 2 with a higher total longitudinal reinforcement ratio of $\rho_{l2}=2.332\%$, we observed an increase in the load-carrying capacity of the beam with truss reinforcement ***T-s2*** by 12% in relation to the load-carrying capacity of the beam with conventional reinforcement ***C-s2***.

The analysis of the load versus displacement curves enabled the assessment of the ductility of the beams. The ductility of the beams is defined as the ratio of the ultimate displacement $\upsilon_{u}$ to the displacement $\upsilon_{p}$ registered at the time instant of the beam stiffness change, which can be interpreted as the beginning of the plastic behavior of the beam.

Table 3 shows the ductility ratio k=υuυp determined for the coordinates of the points on the load-displacement graphs of the tested beams Series 1, Figure 5 and Series 2, Figure 6.

In the beams of Series 1, the beam with truss reinforcement ***T-s1*** shows more than 5.5 times lower ductility ratio compared to that for the beam with conventional reinforcement ***C-s1***. The reason for such behavior is the significant increase in the load-carrying capacity and the delay in the appearance of plastic effects in the beam with truss reinforcement.

In the beams of Series 2, similar ductility was observed both in the beam with the truss reinforcement ***T-s2*** and in the beam with the conventional reinforcement ***C-s2***.

## 6. Results of Beam Cracking Tests

The following groups of drawings present images of the cracking state recorded at selected points $c_{i}\left(Q,v_{A}\right)$ and $t_{i}\left(Q,v_{A}\right)$ marked in the diagrams load-carrying capacity-displacement for beams with conventional reinforcement and with truss-shaped reinforcement, respectively. The focus was on a comparative analysis of the cracking layout and the range of cracks at the height of the cross-section. The figures show the range of cracks, which is described with accuracy to ½ of the grid spacing of measurement points, i.e., 1/14 h, where h=250 mm is the height of the cross-section.

**Figure 7:**
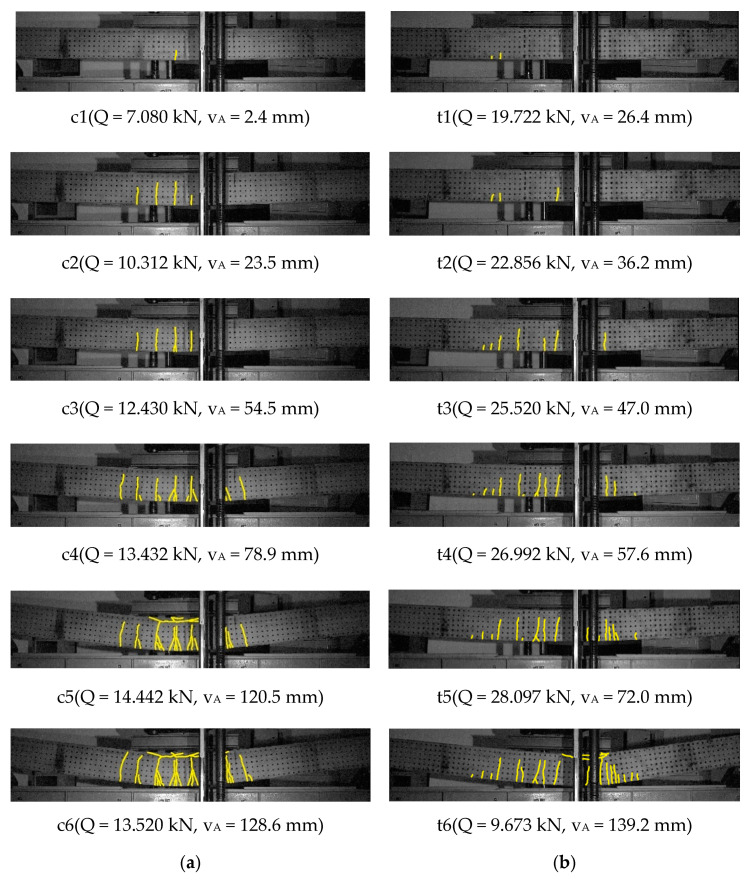
Cracking mechanism of beams of Series 1: (**a**) beam ***C-s1***, (**b**) beam ***T-s1***.

Figure 7 shows the cracking sequences of Series 1 of beams with a low, total longitudinal reinforcement ratio $\rho_{l1}=0.582\%$, for conventional reinforcement ***C-s1***, Figure 7a and for truss reinforcement ***T-s1***, Figure 7b.

**Figure 8:**
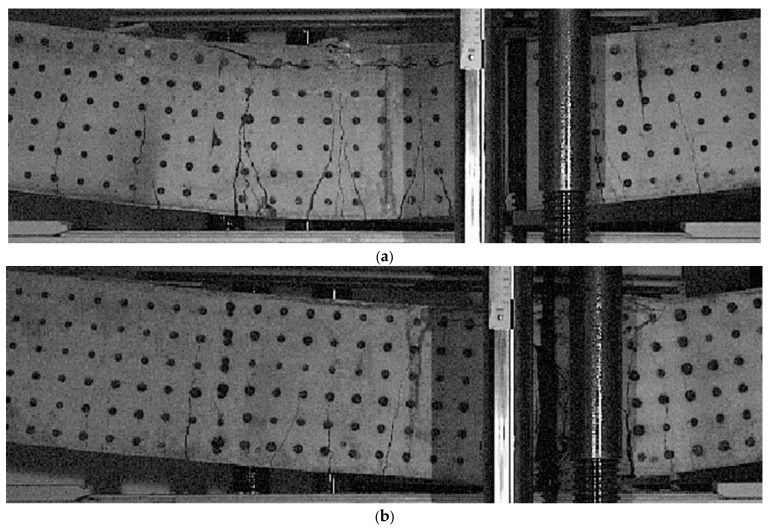
Zoomed view of the final cracking pattern for beams of Series 1: (**a**) beam ***C-s1*** for c6(Q = 13.520 kN, v_A_ = 128.6 mm), (**b**) beam ***T-s1*** for t6(Q = 9.673 kN, v_A_ = 139.2 mm).

Figure 8 shows enlarged images of the cracking state of beams of Series 1 for conventional reinforcement ***C-s1*** for the point c6, Figure 8a and for truss reinforcement ***T-s1*** for the point t6, Figure 8b. The enlargements of the images refer to the pure bending region of the beams.

For the beam with conventional reinforcement, Figure 7a, for the load slightly greater than the calculated cracking load according to Equation (1)_1_—point c1, the first single crack was observed with a range of about 2/7 h.

For the next point c2, with the load exceeding the load causing plasticization of the tensile reinforcement steel, a layout of 4 cracks was observed, out of which the range of the first crack is 5/7 h. Three further cracks reached the ranges of 5/7 h, 4/7 h, and 2/7 h.

For the point c3, at the load exceeding the calculated breaking load according to Equation (1)_2_, the layout of cracks practically did not change, but a secondary crack appeared, connecting with the first crack. The fourth crack reached a range of 4/7 h.

For the point c4, under the load in the range of the load-carrying capacity strengthening, a layout of seven “fan-shaped” cracks over the entire area of pure bending (i.e., in the central area of the beam between two loading forces) was observed, with second and third order cracks connecting to the original cracks, except for the farthest cracks. The spacing of these cracks corresponded to the stirrups spacing. The cracks had almost the same a range of about 5/7 h. The first main crack was accompanied by two secondary cracks.

For the point c5, with the load corresponding to the load-carrying capacity of the beam, the appearance of a longitudinal crack along the bars in the compressed concrete layer in the central part of the pure bending area was observed, together with a range increase of the second and third order cracks. In the vicinity of the first main crack, four secondary cracks were found, one of which reached a range of 5/7 h almost equal to that of the main crack.

For the point c6, at the post-critical load corresponding to the maximum beam displacement and preceding the rapid disintegration of the concrete in the compressed zone, an elongation of the longitudinal crack range along the bars in the compressed concrete layer was observed. An increase in the range of all cracks to about 6/7 h was found. At the moment of beam destruction, the longest secondary crack accompanying the first main crack and the second main crack turned out to be the dominant cracks, which determined the plastic hinge of the beam and caused the unrestrained movement of the beam.

For the beam with the truss reinforcement, Figure 7b, at the load of almost three times greater than the calculated cracking load according to Equation (2)_1_—point t1, asymmetrically—on the left, just beyond the boundary of the pure bending area, the first two initiating cracks with a range of 1/7 h and 2/7 h were observed.

For the next point t2, under the load in the range of the load-carrying capacity strengthening, the alignment of the first two cracks was observed without changing their width. In addition, the third crack appeared in the center of the pure bending region, with a range of 3/7 h.

For the point t3, four consecutive cracks were observed in the pure bending region, with a range from 3/7 h to 5/7 h, and one new crack outside the pure bending region with a range of 1/7 h. The fifth crack under the right force reached a range of 4/7 h.

For point t4, further cracks were observed in the pure bending region, with a range of 4/7 h and 3/7 h, and two new cracks located symmetrically outside the pure bending region, with a range of 1/7 h.

For the t5 point, under the load corresponding to the beam load-carrying capacity, a similar to the previously analyzed “comb-shaped” layout of cracks was observed, with the range gradually increasing towards the center of the beam from 1/7 h to 5/7 h. There were also two new cracks under the right-hand side loading force—a sixth crack with a range of 3/7 h and a seventh crack with a range of 3/14 h.

For the t6 point, at the lowest post-critical load corresponding to the maximum beam displacement and preceding the concrete crushing of the compression zone, the appearance of longitudinal cracks along the reinforcement bars in the compressed concrete layer in the right part of the pure bending region was observed. There were also other transverse cracks beyond the right boundary of the pure bending region with a range of 2/7 h. Almost the same range, 5/7 h, of all cracks inside the pure bending region was observed. The last two cracks under the right loading force reached the range of 4/7 h—crack six and 3/14 h—crack seven. At the moment of beam destruction, the seventh crack turned out to be a dominant one, forming, along with the sixth crack, a plastic joint of the beam under the right-hand side loading force and leading to a rapid disintegration of the compressed concrete zone.

**Figure 9:**
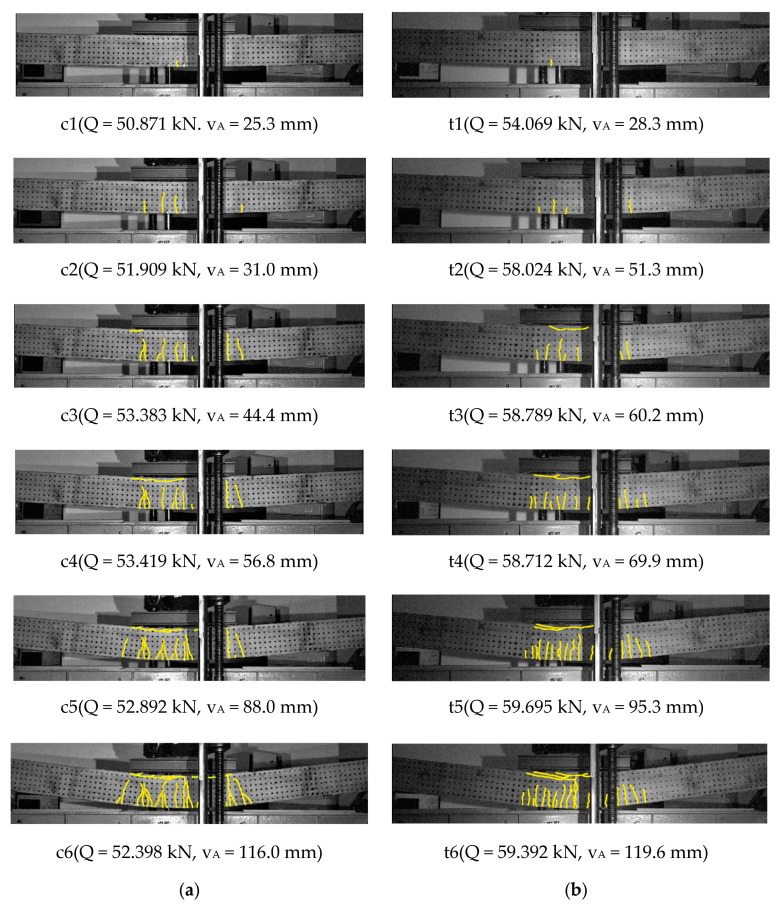
Cracking mechanism of beams of Series 2: (**a**) beam ***C-s2***, (**b**) beam ***T-s2***.

Figure 9 presents subsequent cracking layouts of Series 2 of the beams with a high total longitudinal reinforcement ratio $\rho_{l2}=2.332\%$, for conventional reinforcement ***C-s2***, Figure 9a and for truss-shaped reinforcement ***T-s2***, Figure 9b.

**Figure 10:**
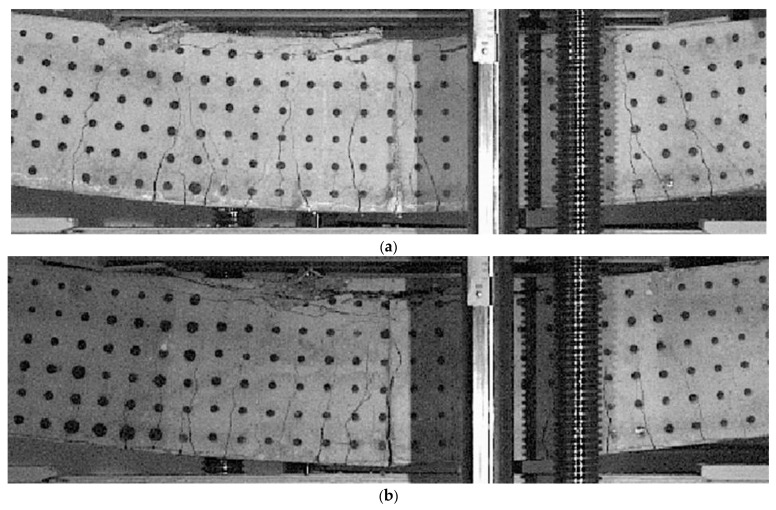
Zoomed view of the final cracking pattern for beams of Series 2: (**a**) beam ***C-s2*** for c6(Q = 52.398 kN, v_A_ = 116.0 mm), (**b**) beam ***T-s2*** for t6(Q = 59.392 kN, v_A_ = 119.6 mm).

In turn, Figure 10 shows enlarged images of the cracking state of beams of Series 2 for conventional reinforcement ***C-s2*** for the point c6, Figure 10a, and for truss reinforcement ***T-s2*** for the point t6, Figure 10b.

For the beam with conventional reinforcement, Figure 9a, for the load almost six times greater than the calculated cracking load according to Equation (2)_1_ and even a value greater than the calculated load-carrying capacity according to Equation (2)_2_—point c1, the first, single crack was observed with a range of about 3/14 h.

For the next point c2, at the load exceeding the load causing plasticization of the tensile reinforcement steel, a layout of 4 cracks was observed, among which the range of the first crack extended to 4/7 h, whereas the range of the longest, the second crack was 9/14 h. The third crack under the left loading force had a range of 3/7 h, and the fourth crack on the outside of the right loading force had a range of 2/7 h.

For the point c3, at the load close to the beam load-carrying capacity, the first longitudinal crack appeared in the cover of the compressive reinforcement, in the short region of the transfer of the left loading force. A layout of 6 main normal cracks was formed: the first one with a range of 4/7 h, the second, third, and fourth ones with a range of 5/7 h, as well as two new cracks: the fifth one almost in the center of the pure bending region ranged 4/7 h, and the sixth one on the inner side of the right loading force with a range of 6/7 h. Secondary cracks appeared close to the second, third, and fifth cracks, joining the main cracks, with lengths of 2/7 h, 7/14 h, and 3/14 h, respectively.

For the point c4, with the load corresponding to the beam load-carrying capacity, the length increasing in the longitudinal crack was observed along the reinforcement bars in the compressive concrete layer in the left part of the pure bending region. The layout and the range of the main cracks hardly changed. However, new secondary cracks appeared at the first and the third main cracks.

For the point c5, under the load in the post-critical range, a further increase in the longitudinal crack length was observed along the reinforcement bars in the compressive concrete layer towards the right part of the pure bending area. There were also new secondary cracks at the second and the fifth main cracks. An inclined seventh main crack appeared on the left edge of the pure bending region with a range of 4/7 h. The rightmost secondary crack at the fifth main crack reached a range of 11/14 h.

For the point c6, with the load preceding the ultimate failure load, the occurrence of a longitudinal crack along the reinforcement bars in the compressive concrete layer was observed in the entire pure bending region. The final layout of the first order main normal cracks was formed, with ranges from 4/7 h to 6/7 h. The “fan-shaped” layout of associate secondary cracks of the second and third order was characteristic. At the moment of beam failure, the fifth main crack (in the center of the pure bending region) and the sixth main crack (under the right loading force) joined the longitudinal crack along the reinforcement bars in the compressive concrete layer. New longitudinal cracks appeared in this zone, causing the concrete to break out in the compression zone and—as a consequence—the unrestrained movement of the beam.

For the beam with truss reinforcement, Figure 9b, for a load of more than six times greater than the calculated cracking load according to Equation (2)_1_ and a value greater than the calculated load-carrying capacity according to Equation (2)_2_—point t1, the first single normal crack was observed with a range of about 3/14 h.

For the next point t2, at the load exceeding the load causing plasticization of the tensile reinforcement steel, a layout of 4 cracks was observed, among which the range of the first crack extended to 3/7 h. The range of the second longest crack under the right loading force was also of 3/7 h. The two remaining cracks, on the left-hand side (third crack) and right-hand side (fourth crack) of the first crack, had a range of 3/14 h.

For the point t3, the longitudinal crack appeared in the axis of the compressive reinforcement, in the central region of pure bending. Furthermore, the layout of 7 normal cracks occurred with the following ranges: the first crack 9/14 h, the second crack 4/7 h, the third crack 3/7 h, the fourth crack 3/14 h together with 3 new cracks: the fifth crack in the center of the pure bending region 3/7 h, the sixth and the seventh cracks under the left and right loading forces of 2/7 h.

For the point t4, with the load preceding the reaching of the beam load-carrying capacity, the length increasing of the longitudinal crack was observed along the reinforcement bars in the compressive concrete layer in the left part of the pure bending region. The ranges equalization of the previous crack layout was observed. The range of the first crack shortened to 3/7 h. The second crack did not change the range. The third and fourth cracks were of 3/14 h ranges. The fifth and the sixth cracks had a range of 3/7 h, and the seventh crack—2/7 h range. The five new cracks appeared with the range from 2/7 h to 3/7 h—three of them in the center part of pure bending region and the two remaining ones in the region beyond the right loading force.

For the t5 point, at the load corresponding to the beam load-carrying capacity, the appearance of a second longitudinal crack along the reinforcement bars in the compressive concrete layer in the left part of the pure bending area was observed. Moreover, a “comb-shaped” layout of single 16 normal cracks was observed, with the range gradually increasing towards the center of the beam from 2/7 h to 5/7 h. The following ranges of cracks were found: the first 4/7 h, the second 5/7 h, the third and the fourth 7/14 h, the fifth 5/7 h, the sixth 3/7 h, and the seventh 7/14 h. The four new cracks reached ranges and widths from 2/7 h (the most left-hand side placed crack) to 4/7 h (cracks between the fourth and the fifth in the center of the pure bending region and between the third and the sixth near the left force loading).

For the t6 point, for the post-critical load preceding the concrete crushing of the compressed zone, the appearance of a third longitudinal crack was observed along the reinforcement bars in the compressive concrete layer in the left part of the pure bending region. Moreover, one new transverse crack was observed beyond the left boundary of the pure bending region with a range of 2/7 h. First of all, the concentration and redistribution of the cracking state inside the central part of the pure bending region was noted. Namely, nearly the same large range of 5/7 h of the fourth crack and the crack next to its right-hand side was observed. Particularly visible was the splitting and widening of the fifth crack dominant, which merged with the longitudinal cracks. The closure of two shorter cracks adjacent to the left-hand side of the dominant crack occurred, including the first crack, which shortened to 2/7 h. External cracks, the third and the sixth under the left loading force as well as the second and the seventh under the right loading force, reached almost the same range of 5/7 h. At the moment of reaching the maximum displacement, the failure mechanism with the dominant transverse crack (crack five) and the delamination of the compressive concrete zone leading to unrestrained beam movement were observed.

## 7. Discussion of Results

### 7.1. General Characteristics and Evaluation of Results

The main goal of study was to compare the load-carrying capacity of beams with possibly the same stiffness over the entire length of the beam. The tests were carried out for a truss reinforcement with a fixed angle between transverse bars. Due to the truss structure of the reinforcement, a higher load-carrying capacity could be expected in the areas where shear forces dominated. Further tests are planned for truss reinforcement with different angles between the transverse bars.

The shear resistance of the beam was not investigated in this study because the beams with a very high shear slenderness ratio (equal to $\lambda_{s}=\frac{c}{d}=$ 4.57, where c and d data—see Appendix A) were tested, but an increase in the load-carrying capacity of the beam with truss reinforcement was demonstrated in the four-point bending test. Thus, the influence of the new reinforcement system on increasing the beam load-carrying capacity was shown. This increasing in the beam load-carrying capacity only indirectly indicates the possibility of increasing the shear resistance. Therefore, a full demonstration of the advantages of the proposed reinforcement system in beam shear testing is planned.

The accuracy of the non-contact optical method used in the study is sufficient to determine the displacement curve as a load function. Therefore, the strains were not monitored in the tests. Only the crack pattern with visualization of the cracks’ ranges at the cross-section height was presented without measuring of the crack width. In subsequent studies, we will consider the use of displacement transducers and strain gauge methods for displacements and strain measuring. First, however, it is planned to develop the optical method so as to increase the accuracy of measurements and the level of automation of processing the large number of results obtained in the continuous registration process. Achieving such accuracy is especially important when determining the time of appearance of very fine cracks (of sub-millimeter size) and measuring the crack width.

The practical implementation of the proposed reinforcement system is planned after a more comprehensive studies of various structural elements. Such structural solutions are presented in detail in the patent description [P1].

### 7.2. Similarities in the Behavior of Series 1 and Series 2 Beams Observed during the Test

The similarities in the behavior of the beams of Series 1 and Series 2 relate primarily to the cracking mechanism. The comparative analysis of the cracking mechanisms presented in Figure 7, Figure 8, Figure 9 and Figure 10 allows the derivation of the following statements.

- The range of perpendicular cracks at the height of the cross-section along the pure bending region is smaller in beams with the truss reinforcement system.
- The region of perpendicular cracks occurrence exceeds the region of pure bending both in the beam with conventional reinforcement and in the beam with the truss reinforcement system.
- A greater number of perpendicular cracks with a smaller spacing and a shorter range over the cross-section height, are observed in the beams with the truss reinforcement system, than in the beam with a conventional reinforcement system.
- The “comb-shaped” crack pattern is in beams with a truss reinforcement system, and the “fan-shaped” crack pattern is in beams with a conventional reinforcement system.

### 7.3. The Specific Features of the Behavior of Series 1 Beams

The comparison of the behavior of the beams of Series 1 with conventional reinforcement and with truss reinforcement shows the following facts.

- Very significant increase in the load-carrying capacity by 95% in the beam with truss reinforcement compared to that for the beam with conventional reinforcement was stated.
- The load-carrying capacity was achieved with displacements of 40% less in the beam with truss reinforcement than for the beam with conventional reinforcement.
- The occurrence of the load-carrying capacity decreasing path after achieving the load-carrying capacity in the beam with truss reinforcement was observed.
- The cracking appearance in the beam with the truss reinforcement system was recorded at almost three times higher load level than in the beam with the conventional reinforcement system, assuming the available image recording accuracy.
- At the load corresponding to the load-carrying capacity in the beam with the truss reinforcement system, no horizontal cracking along the reinforcement bars in the compressive concrete layer was observed.
- The process of post-critical behavior of the beam with the truss reinforcement system is significantly extended and the occurrence of a sudden breakdown of the compressive zone of concrete is not observed even when the maximum displacements of the beam was reached, with a value corresponding to the ratio of the beam span equal to l/21.55.
- More than 5.5 times lower ductility ratio was observed for the beam with truss reinforcement than for the beam with conventional reinforcement; however, the absolute value of the ductility ratio of the beam with truss reinforcement was large and was of about 15% greater than that for Series 2 beams.

### 7.4. The Specific Features of the Behavior of Series 2 Beams

The comparison of the behavior of Series 2 beams with conventional reinforcement and with truss reinforcement indicates the following facts.

- Increase in the load-carrying capacity by 12% in the beam with truss reinforcement compared to that for the beam with conventional reinforcement was stated.
- The load-carrying capacity was achieved with displacements of 68% greater in the beam with truss reinforcement than for the beam with conventional reinforcement.
- The appearance of a crack in a beam with the truss reinforcement system was recorded at a load level about 6% greater than in the beam with the conventional reinforcement system, assuming the available image recording accuracy.
- At the load equal to the load-carrying capacity, both in the beam with the conventional reinforcement and in the beam with the truss reinforcement system, the occurrence of horizontal cracking along the reinforcement bars in the compressive concrete layer was observed.
- The process of post-critical behavior both in the beam with the conventional reinforcement and in the beam with the truss reinforcement system is elongated, but there is a rapid disintegration of the compressive zone of concrete when the maximum displacement of the beam was reached, with a value corresponding to the ratio of beam span equal to about l/25.
- Almost the same ductility ratio was observed both for the beams with truss reinforcement and with conventional reinforcement.

## 8. Conclusions

The paper demonstrates experimental verification of the effectiveness of the proposed innovative truss type reinforcement system for reinforced concrete beams.

The originality of the proposed reinforcement system consists in the truss shaping of diagonal transverse bars and the permanent connection of the reinforcement bars at all connection points. Its main feature is self-supporting and the ability to carry the initial load even before concreting.

The following basic conclusions were derived from our novel experiments on reinforced concrete beams with truss reinforcement.

The most important achievement of the research is proof that the use of the truss reinforcement influences increasing the load-carrying capacity of beams.

The magnitude of load-carrying capacity increase depended on the total longitudinal reinforcement ratio of beams. For beams with a low value of longitudinal reinforcement ratio, the influence of the transverse truss-type reinforcement on load-carrying capacity of beams was considerable. Thus, for the total longitudinal reinforcement ratio equal to 0.582%, the beam with truss reinforcement showed an increase in load-carrying capacity by 95% compared to that of a beam with conventional reinforcement, while for the total longitudinal reinforcement ratio equal to 2.332%, the increase in load-carrying capacity was 12%.

For the beams in Series 1 with a low value of total longitudinal reinforcement ratio, the load-carrying capacity was achieved with displacements of 40% less in the beam with truss reinforcement than for the beam with conventional reinforcement, whereas for the beams in Series 2 with a high value of total longitudinal reinforcement ratio, the load- carrying capacity was achieved with displacements of 68% greater in the beam with truss reinforcement than for the beam with conventional reinforcement. These observations confirm the greater degree of stiffening for the beams with truss reinforcement at a lower longitudinal reinforcement ratio.

The non-contact optical method was used in the study to determine with sufficient accuracy for the displacement measurement to build a displacement curve as a load function. However, such accuracy was not sufficient for measuring the crack width. Therefore, only the crack pattern with visualization of the cracks’ range at the cross-section height could be presented.

Based on the investigation of the cracking mechanism, one can conclude that the failure of beams with transverse truss reinforcement occurs with a greater number of scattered cracks, more evenly distributed over the length of the cracking zone and with smaller crack ranges over the cross-sectional height.

The cracking pattern stated in the research proved once again the stiffening effect of the truss-shaped reinforcement on the effort distribution over a larger part of the beam than in the case of using conventional transverse reinforcement in the form of stirrups perpendicular to the longitudinal axis of the beam.

The authors perceive the potential of the non-contact optical method development in the context of deformation analysis of structural elements, based on the recorded displacements in the set of measurement points marked on the element.

The authors are fully conscious of the need to further develop research in order to confirm the full effectiveness and high potential application possibilities of the new type of truss reinforcement under various loads and in various structural elements.

## 9. Patent

P1. Stolarski, A.; Zychowicz, J. Flat grids system with the lattice arrangement of bars for concrete reinforcement. Patent description PL 226083 B1, 30.06.2017. Number of patent declaration 398305, 05.03.2012. Polish Patent Office. Patent holder Military University of Technology, Warsaw, Poland.

## Figures and Tables

**Table 1:** Compressive strength of concrete.

Beam Series	Sample No.	Failure Load (kN)	Compressive Strength (MPa)			Concrete Density (kg/m^3^)
			$f_{ci}$	$f_{cm}$	$f_{c,min}$	
***s1***	1	1481	65.8	***f_cm,1_* = 65.3**	64.3	2280
	2	1483	65.9			
	3	1440	64.3			
***s2***	1	1442	64.1	***f_cm,2_* = 64.6**	64.1	2350
	2	1451	64.5			
	3	1469	65.3			

**Table 2:** Tensile strength of reinforcement steel.

Beam Series	Bar Diameter (mm)	Yield Limit $R_{e}\;\left(MPa\right)$	Tensile Strength $R_{m}\;\left(MPa\right)$
***s1***	8	541	615
***s2***	16	530	620
***s1***, ***s2***	6	532	620

**Table 3:** Ductility ratio of beams.

Beam Type	Ultimate Displacement $\upsilon_{u}$ (mm)	Yield Displacement $\upsilon_{p}$ (mm)	Ductility Ratio k=υuυp
***C-s1***	c6(Q = 13.520 kN, **v_A_ = 128.6 mm**)	cp(Q = 9.340 kN, **v_A_ = 4.4 mm**) ^1^	29.10
***T-s1***	t6(Q = 9.673 kN, **v_A_ = 139.2 mm**)	t1(Q = 19.722 kN, **v_A_ = 26.4 mm**)	5.27
***C-s2***	c6(Q = 52.398 kN, **v_A_ = 116.0 mm**)	c1(Q = 50.871 kN, **v_A_ = 25.3 mm**)	4.58
***T-s2***	t6(Q = 59.392 kN, **v_A_ = 119.6 mm**)	tp(Q = 53.760 kN, **v_A_ = 26.4 mm**) ^1^	4.53

^1^ coordinates of the points determined, but not marked in the load-displacement diagrams.

## Data Availability

Data sharing not applicable.

## Appendix A. Analytical Values of the Cracking Force and the Failure Force

The analytical values of the cracking force $Q_{cr}$ and the failure force $Q_{0}$ were determined, disregarding the action of the deadweight of the beam, as follows:

(A1) $$Q_{cr}=\frac{M_{cr}}{c},\;Q_{o}=\frac{M_{o}}{c}$$

where $M_{cr}$ and $M_{o}$ are the cracking and failure moments determined in the bending reinforced concrete cross-section, respectively for the linear elastic phase *Ia* and the limit phase *III* with rectangular stress distribution in concrete, see e.g., [4,35]; and c is the distance of the loading force axis from the support reaction force axis, Figure 2.

The cracking moment is determined on the basis of the static equilibrium condition of the elastic equivalent cross-section in the form:

$$M_{cr}=f_{ctm}W_{cs}^{t},$$

where $f_{ctm}$ is the mean tensile strength of concrete and $W_{cs}^{t}$ is the section modulus with respect to the tension extreme layer determined from the relationship/

$$W_{cs}^{t}=\frac{I_{cs}}{h-x}$$

$I_{cs}$ is the second moment of area of concrete section related to the neutral axis, determined from the relationship

$$I_{cs}=\frac{1}{3}bx^{3}+\frac{1}{3}b{\left(h-x\right)}^{3}+\alpha_{e}[A_{s1}{\left(d-x\right)}^{2}+A_{s2}\left(x-a_{2}{)}^{2}\right]$$

As a function of geometrical parameters of the cross-section: overall width b, overall high h, effective high $d=h-a_{1}$, the cross-sectional area of the tensile $A_{s1}$ and compressive $A_{s2}$ reinforcement, located at distances $a_{1}$ and $a_{2}$ from the lower and upper edge of the cross-section, and the neutral axis depth are determined from the equation

$$x=\frac{\frac{1}{2}bh^{2}+\alpha_{e}\left(A_{s1}d+A_{s2}a_{2}\right)}{bh+\alpha_{e}(A_{s1}+A_{s2})}$$

with the proportionality coefficient of the deformation moduli of reinforcing steel and concrete $\alpha_{e}=\frac{E_{s}}{E_{cm}}$.

In turn, the failure moment is determined on the basis of the static equilibrium condition of the reinforced concrete cross-section in the state of the ultimate load-carrying capacity, in the form

$$M_{0}=\{\begin{array}{ccc} bxf_{cm}\left(d-\frac{\bar{x}}{2}\right)+A_{s2}f_{y}z_{a} & if & d-\frac{\bar{x}}{2}\leq z_{a}\\ A_{s1}f_{y}z_{a} & if & d-\frac{\bar{x}}{2}>z_{a} \end{array},$$

where the effective depth of the concrete compressive zone is

$${\bar{x}}_{min}\leq \bar{x}=\frac{(A_{s2}-A_{s1})f_{y}}{bd^{2}f_{cm}}\leq {\bar{x}}_{lim}$$

where limitations ${\bar{x}}_{min}=2a_{2}$ and ${\bar{x}}_{lim}$—according to [35], $z_{a}=d-a_{2}$ determines the lever arm of internal forces in the layers of the tensile and compressive reinforcement bars, and $f_{y}$ is the yield strength of reinforcement.

The values of the cracking force and the failure were determined according to (A1) on the basis of the following data.

Namely, the geometric data were adopted for the calculations in accordance with the Figure 2: c=105 cm, b=15 cm, h=25 cm, for the symmetrical double reinforced cross-section with: $A_{s1}=A_{s2}=\{\begin{array}{cc} 1.01\;{\mathrm{cm}}^{2} & for\;beam\;of\;Series\;1\\ 4.02\;{\mathrm{cm}}^{2} & for\;beam\;of\;Series\;2 \end{array}$, and $a_{1}=a_{2}=2\;\mathrm{cm}$.

In turn, the data on the mean compressive strength of concrete $f_{cm}$ (MPa) were taken from Table 1 and the yield strength of steel $f_{y}=R_{e}$ from Table 2.

However, the mean concrete tensile strength and the mean concrete deformation modulus were determined based on the standard formulae according to [35]:

$$f_{ctm}=0.30\cdot {\left(f_{cm}-8\right)}^{2/3},\;(\mathrm{MPa}),\;\mathrm{and}\;E_{cm\;}=22\cdot {\left(0.1\cdot f_{cm}\right)}^{0.3},\;(\mathrm{GPa}).$$

The value of modulus of elasticity of reinforcing steel was assumed as $E_{s}=205$ (GPa).

As a result, the calculated values of the cracking force and the failure according to (A1) are as follows:

(A2) $$\mathrm{for}\;\mathrm{beam}\;\mathrm{of}\;\mathrm{Series}\;1:\;Q_{cr1}=6.87\;\mathrm{kN}\;i\;Q_{01}=10.88\;\mathrm{kN},$$

(A3) $$\mathrm{for}\;\mathrm{beam}\;\mathrm{of}\;\mathrm{Series}\;2:\;Q_{cr2}=8.00\;\mathrm{kN}\;i\;Q_{02}=42.63\;\mathrm{kN}.$$
